# Contemporary Clinical Utilization of Radioembolization with Immune Checkpoint Inhibitors as First-Line Treatment in HCC: Real-World Report on Safety and Outcomes

**DOI:** 10.3390/cancers17172745

**Published:** 2025-08-23

**Authors:** Kelley G. Núñez, Tyler Sandow, Alexandre Grahovac, Ricardo Vallejo-Calzada, Juan Gimenez, Humberto Bohorquez, Ari Cohen, Jonathan Mizrahi, Lingling Du, Paul Thevenot

**Affiliations:** 1 Institute of Translational Research, Ochsner Health System, New Orleans, LA 70121, USA; 2 Interventional Radiology, Ochsner Health System, New Orleans, LA 70121, USA; 3 Ochsner Clinical School, University of Queensland, New Orleans, LA 70121, USA; 4 Department of Hematology and Oncology, Ochsner Clinic Foundation, New Orleans, LA 70121, USA; 5 Multi-Organ Transplant Institute, Ochsner Health System, New Orleans, LA 70121, USA; 6 Faculty of Medicine, University of Queensland, Brisbane, QLD 4072, Australia

**Keywords:** immune checkpoint inhibitors, hepatocellular carcinoma, Yttrium-90

## Abstract

Immune checkpoint inhibitors (ICIs) have emerged as first-line therapy for advanced-stage HCC with modest response rates (<33%). Combination treatments offer the potential to improve response rates while improving outcomes. This study evaluates the safety and outcomes of first-line Yttrium-90 plus ICI (^90^Y-ICI). A retrospective, multi-center study was conducted in HCC patients receiving first-line ^90^Y-ICI with atezolizumab plus bevacizumab (Atezo/Bev) or tremelimumab plus durvalumab (Treme/Durva). Treatment response was evaluated at follow-up for planned ^90^Y treatment cycle. Time-to-event measures of time to progression (TTP), progression-free survival (PFS), and overall survival (OS) served as primary endpoints, with first cycle response rates and AEs as secondary outcomes. This study included 37 patients receiving ^90^Y-ICI from 2021 to 2024 (16-month median follow-up). The cohort was predominantly Child-Pugh A (92%) with HCV-related cirrhosis (67%), advanced stage (54%), and a median index HCC size of 8.0 cm (IQR: 6—12 cm). Grade 3–4 AEs occurred in six patients (16%). The target objective response (OR) rate was 83%, with a 50% target complete response (CR) rate. Overall OR was 61% with an overall CR of 39%. Median PFS was 11 months with 1-year rates of 50%. Patients with a target CR had improved TTP (*p* = 0.004), PFS (*p* = 0.003), and OS (*p* = 0.003). The cohort’s 2-year OS was 41% with a median OS of 19 months (CI: 12–37 months). First-line ^90^Y-ICI therapy in HCC is safe and effective, with no deviation in anticipated results. Patients achieving target CR showed significantly improved TTP, PFS, and OS, supporting target CR as an optimal treatment target.

## Simple Summary

Immune checkpoint inhibitors (ICIs) serve as first-line therapy for advanced-stage hepatocellular carcinoma but continue to have modest treatment response rates (<33%). Clinical trials are underway that combine Yttrium-90 plus ICI (^90^Y-ICI) to combat low response rates from ICI therapy alone. In this retrospective, multi-center study, contemporary clinical utilization of ^90^Y-ICI demonstrated high target objective and complete response rates of 83% and 50%, respectively. Prolonged time to progression, progression-free survival, and overall survival were achieved in patients that achieved a target complete response with limited grade 3–4 adverse events (16% of patients). These findings provide a real-world assessment of the safety and impact of outcomes with ^90^Y is combined with ICIs.

## 1. Introduction

Hepatocellular carcinoma (HCC) is the fourth leading cause of cancer-related deaths worldwide [1], with incidence rates expected to exceed 1 million in 2025 [2]. The treatment landscape in advanced-stage HCC has dramatically changed in the last 5 years, with immune checkpoint inhibitors (ICIs) supplanting tyrosine-kinase inhibitors (TKIs) as first-line therapy. Initially, ICI monotherapy trials including CheckMate 459 (nivolumab) and KEYNOTE-240 (pembrolizumab) showed improved response rates relative to TKIs, sorafenib and lenvatinib but did not translate to an overall survival benefit (OS) [3,4]. ICI/biologic (anti-VEGF) and ICI combination therapy in the IMbrave150 (atezolizumab and bevacizumab) and HIMALAYA (tremelimumab and durvalumab), respectively, yielded improved objective response rates (ORRs) and OS outcomes compared to sorafenib [5,6,7,8]. These results led to the Atezo/Bev and Durva/Treme regimes being established as first-line treatment options for advanced HCC. Although several other ICI combinations have been investigated (ORIENT-32, COSMIC-312, LEAP-002, CARES-310), ORRs remain low (15–36%) [5,6,9,10,11,12], and despite these advancements, anticipated OS outcomes in advanced-stage HCC remain dismal.

The most promising current strategy to improve ORRs with ICIs is combination liver-directed therapy (LDT). Currently, LDT is used in early- to intermediate-stage HCC to bridge or downstage to surgical treatment options (resection or liver transplantation) and can also serve as a definitive treatment option in some patients. The Barcelona Clinic Liver Cancer (BCLC) staging and treatment algorithm currently includes transarterial chemoembolization (TACE), transarterial radioembolization (TARE), and microwave ablation as LDT options based on tumor size, burden of disease, and preserved liver function. With an objective response to LDT, tumor burden is reduced through an array of cell death mechanisms, which hypothetically address two key barriers to ICI effectiveness, the immunosuppressive microenvironment and limited tumor neoantigens. By addressing these barriers early in the treatment timeline, combination therapy provides a rationale to improve initial ORRs and potentially extend durable complete response rates, leading to improved OS. Several initial clinical trials utilized TACE in combination with ICIs (reviewed in [13]). EMERALD-1 examined TACE in combination with either durvalumab and bevacizumab or placebo and demonstrated an improved ORR of 43.6%, which translated to a PFS benefit [median PFS 15 months] in primarily BCLC A-B patients [14]. While EMERALD-1 confirmed safety and efficacy with OS, data are still maturing.

Yttrium-90 radioembolization (^90^Y) is established as a safe and effective LDT option for BCLC A-B disease and recommended for treating large, solitary disease in the BCLC staging algorithm [15,16,17,18]. More recently, ^90^Y utilizing personalized dosimetry has provided a more reliable approach to achieve ablative dosing and led to improved ORR in solitary [18] and advanced-stage HCC [19]. There is no clear relationship between ICI target expression and treatment effectiveness, and, thus, identifying a treatment responder population for ICI remains challenging. Recent studies have shown that ICI targets are upregulated post-^90^Y [20,21,22] and may play a critical role in restoring an immunosuppressive tumor microenvironment. ICI therapy can help abrogate this response and potentially extend durable response rates while promoting antitumoral immunity. However, safety and efficacy data with combination ^90^Y-ICI are limited. This real-world study aimed to add to the limited available data on safety and progression-free survival (PFS)/overall survival (OS) outcomes in patients treated with ^90^Y-ICI sequential therapy as well as the potential emerging roles of treatment regime and treatment sequencing in combination therapy.

## 2. Materials and Methods

### 2.1. Study Population and Clinical Data Collection

This retrospective, single-center study with all patients proceeding through a single institutional multi-disciplinary HCC board and was performed with the approval of Ochsner Health’s Institutional Review Board (protocol #2023.172). Inclusion criteria were (i) nonresectable HCC, (ii) non-transplantable HCC, (iii) Child-Pugh score A5–B8, (iv) ECOG score 0 or 1, (v) with an initial treatment plan including ^90^Y in combination with ICI therapy utilizing Atezolizumab (Genentech, San Francisco, CA) plus Bevacizumab (Genentech, San Francisco, CA, USA) (Atezo/Bev) or Tremelimumab (AstraZeneca, Cambridge, UK) plus Durvalumab (AstraZeneca, Cambridge, UK) (Treme/Durva), and (vi) >18 years old. Exclusion criteria included (i) BCLC stage D, (ii) ALBI grade 3, (iii) ECOG-2, (iv) prior LDT treatment, (v) prior systemic therapy, and (vi) patients with disease recurrence after prior surgical intervention. Patients who met criteria from January 2021 to May 2024 were included in this study with the goal of obtaining >1-year median follow-up. General demographics, cirrhosis history and staging, Child-Pugh score, laboratory values, and tumor characteristics were extracted from the electronic medical record.

### 2.2. Immune Checkpoint Inhibitor Regimen

Patients received the following ICI regimens: (i) Atezo/Bev both administered IV with Atezo at 1200 mg every 3 weeks and Bev at 15 mg/kg of body weight, (ii) Treme/Durva both administered via IV according to STRIDE [single-dose Treme at 300 mg plus 1500 mg of Durva every 4 weeks [5]. Bev was withheld from patients receiving ^90^Y until radioembolizations were completed (between 1 and 7 months following ^90^Y). Single-agent Atezo was given to 9 patients, either due to excellent response rates with ^90^Y, uncontrolled hypertension, additional radioembolization, or decompensated liver disease. Chest and abdomen imaging was assessed every 3 months.

Adverse events (AEs) were recorded for both ICI and ^90^Y therapy until discontinuation of ICI or death. AEs were assessed every 3 weeks (Atezo/Bev) or 4 weeks (Treme/Durva) after each ICI cycle and monitored until lost to follow-up, death, or discontinuation of ICI. AEs were graded per the National Cancer Institute Common Terminology Criteria for Adverse Events version 4.03.

### 2.3. Yttrium-90 Radioembolization

All participants received 90Y using Therasphere glass microspheres (Boston Scientific Corporation, Marlborough, MA, USA). Treatment followed the standard two-phase process. Phase one involved a mapping angiogram with Technetium-99 m-labeled macroaggregated albumin infusion to determine tumor vascular supply, perfusion volumes, and verify lung shunt fraction (LSF) with estimated lung dose < 30 Gy. Cone beam CT was performed during the planning angiogram to determine vascular supply to the tumor, confirm complete tumor coverage, and develop a radiation segmentectomy dosing plan with a goal of >205 Gy to the perfused volume according to Committee on Medical Internal Radiation Dose model and consensus guidelines for glass microspheres. The second phase involved radiation segmentectomy by ^90^Y microsphere infusion through selective branches of the hepatic artery previously determined from mapping angiography. Three patients bridged to ^90^Y with TACE either due to high LSF (>15%) or infiltrative HCC.

Contemporary clinical utilization of ^90^Y-ICI was under the direction of the multi-disciplinary tumor board in scenarios to improve response rates for intrahepatic disease while preserving or improving candidacy for full ICI regimes. The criteria included the following: (i) BCLC-A patients with large solitary burden where combination therapy was used to improve treatment response and reduce the risk of out-of-field progression to potentially preserve surgical treatment options and (ii) BCLC-B and C patients to improve response rates and allow for treatment of localized tumor burden. All ^90^Y procedures were performed by a pool of providers.

### 2.4. Study Outcomes

The primary endpoints were time to progression (TTP), PFS, and OS. TTP was defined as the time from initiation of therapy until disease progression based on radiographic imaging from baseline according to the modified RECIST resulting in progression to second-line systemic therapy or enrollment in palliative care. PFS was defined as the time from initiation of therapy (ICI or ^90^Y) until transition to second-line systemic therapy due to radiological progression, palliative care, or death of any cause. OS was defined as the time from initiation of therapy until death of any cause. Secondary endpoints were target and overall response to therapy per modified RECIST and were assessed post-^90^Y. Modified RECIST was categorized as either complete response (CR) or objective response (OR) that included complete and partial responses.

### 2.5. Statistical Analysis

Continuous data were reported as median with interquartile range (IQR), or for treatment dates and cycles, the range. Dichotomous data were reported as the number and percentage of either the total population or the defined subgroup of interest. mRECIST scores were the only variable with missing data due to loss to follow-up or death. Patients with missing mRECIST scores were excluded from statistical analysis, which required an mRECIST score with the excluded number reported in linked tabular data. Dichotomous data were evaluated using the Chi square test, while differences in continuous data among groups were determined using the Mann–Whitney test. Treatment sequencing was evaluated irrespective of treatment regime to maintain subgroup levels above the 20% threshold for Chi square analysis. TTP, PFS, and OS were plotted using Kaplan–Meier curves with between-group differences evaluated using the Log-Rank test. Statistical analysis was performed using JMP Pro 18 with graphical output generated using GraphPad Prism 10.

## 3. Results

### 3.1. Study Cohort

This retrospective study included 37 patients who underwent sequential treatment with ^90^Y and ICI (^90^Y-ICI) between January 2021 and May 2024. Patient demographics are displayed in Table 1. The cohort had a median age of 64 years, where the majority were male (81%), Caucasian (40%), and had a primary liver disease etiology of hepatitis C virus (54%). Child-Pugh score A made up 92% of the cohort and was either A5 (30%) or A6 (62%). The cohort was 54% BCLC-C, followed by 27% BCLC-B, and 19% BCLC-A with 54% having solitary disease. The median index tumor size was 8.0 cm. Within the BCLC-C subgroup, 45% (9/20) had extrahepatic disease and 70% (14/20) had macrovascular invasion.

### 3.2. ^90^Y and ICI Treatment Sequence

A breakdown of the treatment regime and sequencing for combination ^90^Y-ICI is included in Table 2. The most frequent ICI regime plan was atezolizumab plus bevacizumab (Atezo/Bev) (30/37, 81%) with a median of 10 cycles of atezolizumab and 4 cycles of bevacizumab with 10 patients (10/31, 32%) ultimately receiving Atezo monotherapy. The median duration of treatment for Atezo/Bev was 8 months (IQR: 4–16 months). The remaining 19% (7/37) were treated with tremelimumab plus durvalumab (Treme/Durva). A single dose of tremelimumab was administered with a median of nine cycles of durvalumab. The median duration of treatment for Treme/Durva was 13 months (IQR: 6–18 months). Similar discontinuation rates were observed for both ICI therapies (Atezo/Bev: 47%, Treme/Durva: 43%). A total of seven patients switched to a different systemic therapy due to disease progression.

All ^90^Y treatments were planned with personalized dosimetry for radiation segmentectomy. The median target dose to volume was 472, Gray with 30% patients requiring a staged treatment plan to treat the entire tumor burden (Table 2). Four patients required an initial TACE treatment as a bridge to ^90^Y. The most common treatment sequence was initiated by ICI followed by ^90^Y in 86% (32/37) of patients with radioembolization performed at a median of 48 days following first cycle ICI. In 14% (5/37) of patients, the treatment plan was initiated by ^90^Y, with first cycle ICI beginning at a median of 18 days after radioembolization. The frequency of treatment sequence plans was similar between ICI regimes (ICI initiation sequence for Atezo/Bev: 87% and Treme/Durva: 86%).

### 3.3. Safety and Adverse Events

AEs associated with either ^90^Y or ICI treatment were assessed over the duration of treatment and are summarized in Table 3. AEs of any grade were experienced by 89% (33/37) of the cohort and were predominantly attributed to an ICI treatment cycle (ICI: 81% vs. ^90^Y: 54%). AEs that led to steroid use (8/37, 22%), treatment delays (9/37, 24%), or discontinuation of ICI (4/37, 11%) were limited. All grade 3–4 AEs were ICI-mediated. Despite a longer duration of treatment, grade 3–4 AEs were less common in Treme/Durva compared to Atezo/Bev (0% vs. 16%). However, AEs that led to delays in treatment (13% vs. 43%) and steroid use (17% vs. 43%) were more common in patients treated with Treme/Durva compared to Atezo/Bev. The most reported grade 1–2 AE for both ICI and ^90^Y was fatigue, while the most common ICI-mediated grade 3–4 AE was bowel perforation (5%, 2/37) (Supplemental Table S1). While all patients received combination therapy, no AE-related deaths were reported. Neither ICI regimen or treatment sequence impacted the frequency of AEs, treatment delays, or discontinuations (Supplemental Table S2).

### 3.4. Response Rates and Outcomes Following ^90^Y-ICI

The impact of response rates and overall outcomes in supporting the clinical rationale for combining therapy was investigated using the mRECIST and time-to-event outcomes. Response rates following the ^90^Y treatment cycle could be assessed in 97% (36/37) of the cohort and are included in Table 4. A target CR was obtained by 50% of the cohort, with 83% achieving a target OR. Overall CR and overall ORR were 39% and 61%, respectively. Response rates were similar between ICI regimes, although both target and overall response outcomes trended higher in the Treme/Durva regimen (Supplemental Table S3). Treatment sequence was not associated with changes in target or overall response rates.

**Figure 1:**
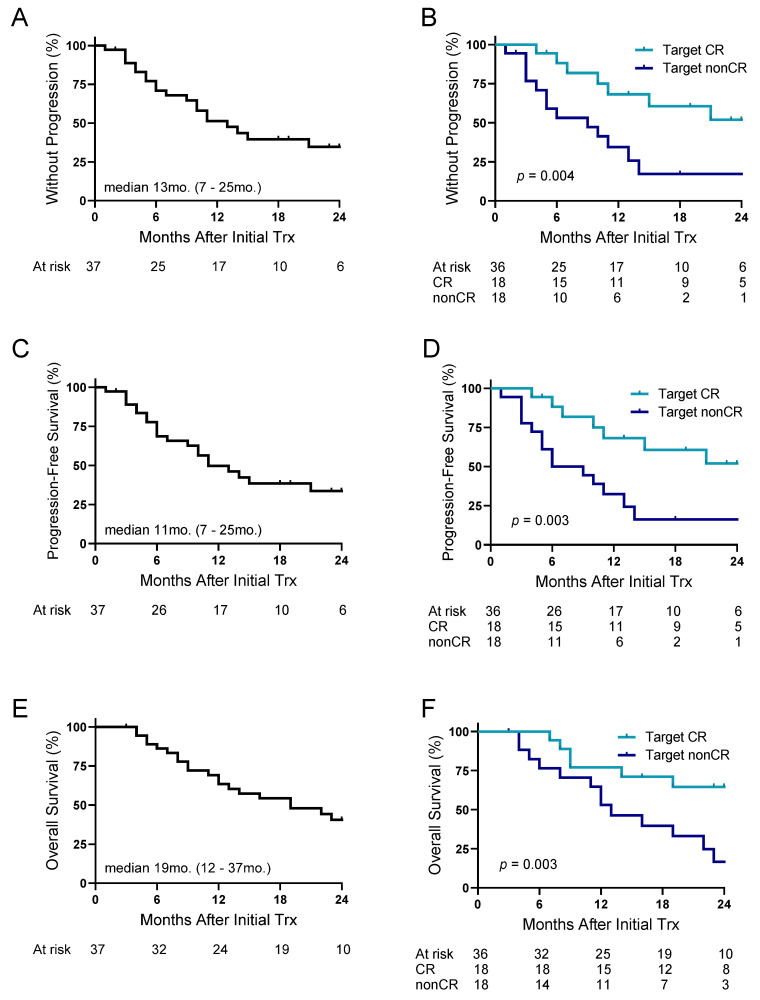
TTP, PFS, and OS following first-line ^90^Y-ICI in HCC. (**A**) Overall TTP and (**B**) TTP based on target complete response rate following initial treatment. (**C**) Overall PFS and (**D**) PFS based on target complete response rate following initial treatment. (**E**) Overall survival and (**F**) OS based on target complete response rate following initial treatment. TTP = time to progression; PFS = progression-free survival; OS = overall survival; CR = complete response.

The median overall follow-up for the cohort was 16 months after the initiation of treatment plan. Overall median TTP was 13 months, with 6-month and 1-year progression rates of 31% and 49%, respectively (Figure 1A). Patients who achieved a target CR following ^90^Y, regardless of sequence regimen, had longer TTP at 6 months (88% vs. 53%), 1 year (68% vs. 34%) and 2 years (52% vs. 17%) compared to those without an initial target CR (Figure 1B). Median TTP was not reached in patients with a target CR compared to 9 months in the nonCR group (CI: 3–13 months). The median PFS was 11 months, with 6-month and 1-year PFS rates of 69% and 50%, respectively (Figure 1C). A similar trend to improved outcomes was observed for PFS in patients with a target CR compared to nonCR at 6 months (88% vs. 50%), 1 year (68% vs. 32%), and 2 years (52% vs. 16%) with median PFS not reached in the target CR group and 7.5 months (CI: 4–13 months) in the target nonCR group (Figure 1D). Median OS for the cohort was 19 months, with 6-month and 1-year OS rates of 86% and 63%, respectively (Figure 1E). The 2-year survival rate was greater than 50% in the target CR group (64% vs. 17% target nonCRs), with median time for OS not reached in the target CR group and 13 months (CI: 6–22 months) in the target nonCR group (Figure 1F).

### 3.5. ^90^Y-ICI in BCLC-C Disease

In BCLC-C disease, where outcomes are more dismal, combination therapy may provide a benefit and improve response rates. Stratifying the cohort to isolate BCLC-C disease revealed that the HCC burden was well controlled between BCLC stages but was accompanied by a higher AFP at diagnosis in BCLC-C patients (median AFP 598 vs. 41 ng/mL) (Table 5). Although the overall burden was well controlled, the ^90^Y target dose to volume trended high in BCLC-C disease and was accompanied by a smaller median perfused volume. BCLC-C patients also had a higher prevalence of HCV as the underlying etiology of liver disease (95% vs. 59%), but all measures of preserved liver function (Child-Pugh, albumin, and bilirubin) remained well controlled. The ICI treatment regimen was well controlled between staging subgroups, with all BCLC-C treatment plans initiated with ICI prior to ^90^Y (100% vs. 71%). Total AEs, severe AEs as well as AEs that led to discontinuation, delays in treatment, or steroid use did not differ between BCLC stages (Supplemental Table S4). The ICI discontinuation rate was notably higher in BCLC-C patients receiving Atezo/Bev (75% vs. 14%) and accompanied by a shorter treatment duration (median: 6 months vs. 10 months). Despite advanced disease, target and overall response rates were similar between staging subgroups (Supplemental Table S4).

**Figure 2:**
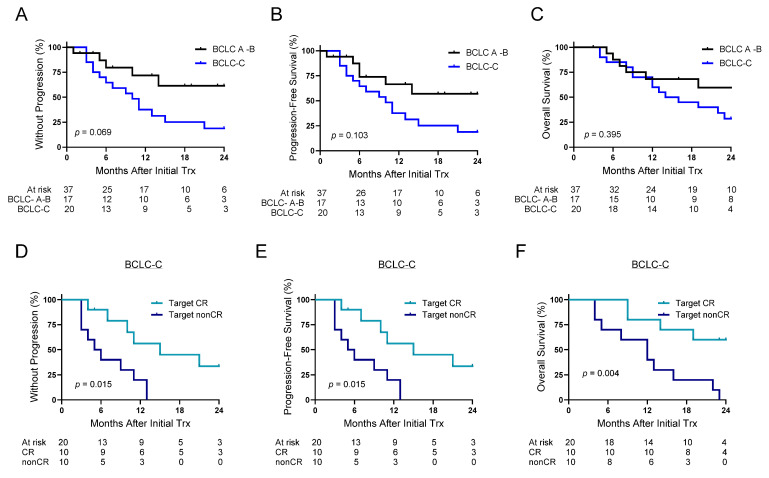
Outcomes based on BCLC stage and Target CR rates in BCLC-C patients. (**A**) TTP, (**B**) PFS, and (**C**) OS based on BCLC A-B versus BCLC-C patients following initial treatment of combination therapy with ^90^Y-ICI. (**D**) TTP, (**E**) PFS, and (**F**) OS in BCLC-C patients based on target CR rates. TTP = time to progression; PFS = progression-free survival; OS = overall survival; Barcelona Clinic Liver Cancer = BCLC; CR = complete response.

The time-to-event outcomes between BCLC A-B and BCLC-C stages were not significantly different (Figure 2). Though TTP was not significantly different, longer TTP was observed for BCLC A-B patients (median 25 months vs. 10 months) (Figure 2A). Similarly, median PFS for BCLC A-B stages was 25 months compared to 10 months for BCLC-C, though not different between the groups (Figure 2B). OS was similar between the two groups (Figure 2C), with median OS of 30 months for BCLC A-B and 15 months for BCLC-C stage. The time-to-event outcome benefits associated with a target CR was most prominent in BCLC-C disease (Figure 2D–F). The target CR rate impacted both TTP and PFS for BCLC-C disease. Significantly higher progression rates were observed for patients with a nonCR with 6-month rates of 60% vs. 10% in those that achieved a target CR (median 5.5 months vs. 15 months) (Figure 2D,E). BCLC-C patients who achieved a target CR had superior OS outcomes compared to target nonCR responders (1-year OS rate: 80% vs. 40%), while the median OS was not reached in CR responders compared to 12 months in nonresponders (Figure 2F).

## 4. Discussion

Real-world data on first-line ICI for HCC continue to show a pronounced survival benefit in the treatment responder population [6,23]. However, only a fraction of patients with advanced HCC are treatment responders, evidenced by low ORRs (20–33%) and even lower CR rates (<8%). Clinical trials have shifted to combination therapy, utilizing various ICI regimens combined with LDT to potentially expand the responder population and improve outcomes by increasing initial response rates. While ^90^Y is gaining momentum as the LDT modality of choice for combination therapy [24], many ^90^Y-ICI clinical trials are still in the enrollment phase, with limited data on the safety, efficacy, and patient outcomes. Recent outcome data from TACE-ICI and retrospective ^90^Y-ICI continue to support the potential in pursuing combination therapy with the hopes of defining a treatment responder population.

LEAP-012 (TACE with Lenvatinib/Pembrolizumab) and EMERALD-1 (TACE with Durva/Bev) were the first clinical trials to investigate LDT-ICI combination therapy. LEAP-012 was able to confirm an ORR and PFS benefit compared to TACE alone, increasing ORR from 33% to 47% and extending median PFS from 10 months to 14.6 months [25]. While LEAP-012 confirmed the potential of ICI to access a greater responder pool in patients still amendable to LDT, EMERALD-1 focused on the ability of TACE to improve the ICI responder pool with Durva alone or plus Bev compared to TACE alone. EMERALD-1 showed ORR increased from 30% to 44% and improved median PFS from 8.2 months to 15 months when TACE was combined with Durva/Bev [14]. There were differences in grade 3 or 4 AEs, with 71% (LEAP-012) and 27% (EMERALD-1), and treatment-related AEs led to discontinuation rates of 8% (LEAP-012) and 28% (EMERALD-1). Both trials heavily favored BCLC A-B disease and supported the safety and efficacy of adding ICIs to LDT in earlier-stage disease. Safety and efficacy in the BCLC-C population, where ICI response rates are low and OS projections still dismal, remain unclear.

^90^Y provides an intriguing alternative to TACE, as it is now established as a safe and effective treatment option for BCLC A-C disease and capable of generating ORRs above 80% [15,18,20,26]. Advancements in personalized dosimetry have further improved outcomes for BCLC-C patients [19], further supporting ^90^Y as a viable LDT choice for combination ICI. Our group and others have shown that ^90^Y elicits a robust immune response [20,22] that, when combined with ICIs, could augment antitumoral immunity by increasing initial response rate and extending the duration of response. The initial retrospective literature on ^90^Y-ICI is promising. A retrospective review of the National Cancer Database identified 142 patients who received ^90^Y plus immunotherapy from 2017 to 2019 [27]. In patients with BCLC B-C disease, combination therapy improved OS compared to immunotherapy alone (19.8 months vs. 9.5 months) [27], which was independently confirmed in a small, single-center study [28]. More recently, the SOLID trial tested the safety and efficacy of combined lobar ^90^Y with single-agent Durva and reported improved ORR (83%) and CR rates (29%) [29], exceeding response rates from EMERALD-1 with TACE-Durva/Bev. High response rates and improved PFS/OS were also confirmed in an eight-patient study using ^90^Y-Atezo/Bev [30]. Additionally, a trial was conducted utilizing ^90^Y in combination with second-line nivolumab for patients with contraindications or tolerance issues with first-line ICIs [31], although with inferior ORRs (<42%). While early evidence supports ^90^Y-ICI, the studies uniformly lack critical details in the treatment algorithm that hinder reproducibility, particularly ^90^Y characteristics (resin vs. glass microspheres, personalized vs. standard dosimetry, segmentectomy vs. lobar) that directly impact response rates and overall outcomes.

In this real-world cohort of predominately BCLC-C (54%), ^90^Y-ICI combination achieved excellent overall and target CR rates (39% and 50%) and ORRs (61% and 83%), regardless of treatment sequence, and resulted in a median PFS of 11 months and OS of 19 months. Patients who achieved a target CR had superior outcomes, with BCLC-C patients with a CR having median PFS of 15 months and 3-year OS rate of 60%. Disease staging is a critical prognostic factor, which hampers direct comparisons of overall effectiveness between ^90^Y-ICI and ICI. Many centers are increasingly utilizing combination therapy in nonsurgical BCLC A-B as an aggressive treatment plan and potential downstage to surgery. This is reflected by the lower percentage of BCLC-C disease in the current study (54%) compared to the hallmark Atezo/Bev (IMbrave150) and Treme/Durva (HIMALAYA) studies, which had 80% and 82% BCLC-C disease, respectively. Additionally, well-selected patients with stable Child-Pugh B disease retain candidacy for combination therapy in the real-world setting compared to the hallmark trial selection criteria. Despite these differences, median OS was similar between ^90^Y-ICI (19 months) compared to Imbrave150 (19.2 months) and HIMALAYA (16.4 months). Additionally, ^90^Y-ICI generated a longer median PFS of 11 months compared to 6.9 months [6], suggesting ^90^Y helped delay tumor progression. Both clinical trials utilized RECIST 1.1 as the primary method to assess treatment response. For this study, tumor response was evaluated using mRECIST, which was developed to assess response following LDT accounting for tumor necrosis [32] and showed superior overall ORR and CR rates of 83% and 50%.

While IMbrave150 and HIMALAYA showed ICI provided an OS benefit compared to sorafenib, another major improvement was the extended duration of response/disease control rate in treatment responders compared to sorafenib [6,8]. In this real-world cohort, with patients with an initial CR, ^90^Y-ICI further improved PFS/OS outcomes (median not reached) compared to those with incomplete responses. The CR effect also extended to patients with BCLC-C disease despite an increased prevalence of MVI (14/20) and extrahepatic disease (9/20), a shorter duration of ICI treatment (Atezo/Bev: 6 months BCLC-C vs. 10 months BCLC A-B; Treme/Durva: 11 BCLC-C vs. 16 months BCLC A-B), and higher ICI discontinuation rates (Atezo/Bev: 75% BCLC-C vs. 14% BCLC A-B; Treme/Durva: 50% BCLC-C vs. 33% BCLC A-B). Within patients with BCLC-C disease, no differences in PFS or OS were observed stratified based on macrovascular invasion versus extrahepatic metastasis ± macrovascular invasion. The next major hurdle is identifying the treatment responder subgroup prior to initiating the treatment plan. Ideally, these biomarkers would retain their prognostic value in both newly diagnosed BCLC-C as well as patients with postsurgical BCLC-C recurrence and progression to BCLC-C from BCLC A-B. These treatment responder profiles may help advance oncogenic profiling and expand treatment opportunities to more precisely target nonresponsive, advanced HCC to improve survival outcomes.

^90^Y and ICI monotherapy have established individual safety profiles, with the limited available data suggesting no detrimental changes in safety profile when using combination therapy. In this study, there were no serious AEs (grade 3 or higher) attributable to the ^90^Y treatment. With real-world applications likely to extend to Child-Pugh B, it will be critical to ensure combination therapy maintains a stable safety profile. Mirroring the Imbrave150 and HIMILAYA trials, this ^90^Y-ICI cohort was predominantly Child-Pugh A and displayed the same common AEs, rash (32%) and fatigue (30%), reported in those trials. Combination therapy was well tolerated, regardless of ICI regimen, with median treatment duration of 8 months for Atezo/Bev (8.4 months for IMbrave150) and a longer Treme/Durva duration of 13 months (5.5 months HIMALAYA). Notably, there were no instances of AE-related mortality. Only two patients experienced any ^90^Y-associated AEs leading to ICI treatment delay, although both resolved post-^90^Y, after which ICIs were resumed.

Although mounting evidence supports ^90^Y-ICI as a safe and effective treatment option, there is no consensus on the optimal patient population, ICI regimen, or treatment sequence. Both current approved first-line regimens target PD-L1, with secondary targets of VEGF with Atezo/Bev and CTLA-4 with Treme/Durva. Currently, the ICI timing and sequence protocols are left to provider discretion, with substantial variance in both the literature and among active clinical trials. While ICI randomized trials may be a future consideration, the available data do not suggest a superior regimen or treatment sequence. Both the ROWAN (NCT05063565) and EMERALD-Y90 (NCT06040099) trials begin their treatment sequence with ^90^Y followed by Treme/Druva (ROWAN) or Durva/Bev (EMERALD-Y90) 2 weeks after treatment. Leading the sequence with ^90^Y will ideally reduce tumor burden (reducing the immunosuppressive impact of the tumor microenvironment) and potentially create a pool of tumor neoantigens prior to ICI administration to increase the likelihood of a productive antitumoral response. Conversely, ICI therapy can cause tumor bulking, which may increase the efficacy of ^90^Y in addition to the ^90^Y-dependent immunological benefits. It will also be critical to evaluate the role of bevacizumab delay in multicycle ^90^Y and patient outcomes as the clinical data pool expands. Adoption of ^90^Y-ICI as a first-line therapy would require robust, multi-center clinical trials that demonstrate safety and efficacy as well as providing guidelines for ^90^Y dosing, ICI regimens, and treatment sequence to facilitate reproducibility.

This study has several limitations. This study is a single-center, retrospective analysis of prospectively collected data grouped according to ^90^Y-ICI treatment regime and sequence, with the goal of adding to the growing body of literature on combination therapy. In our center’s experience, ^90^Y-ICI has been initially utilized as an aggressive approach to treat nonsurgical BCLC A-C disease amendable to ^90^Y with the goal of definitively treating the intrahepatic disease. While this study focused more broadly on safety and efficacy, a propensity score-matched study on nonsurgical BCLC A-B patients treated with ^90^Y vs. ^90^Y-ICI will need to be conducted to address confounding and selection bias. During the early adoption period (2021–2022), there was variance in the preferred ICI regimen due to later approval of the Treme/Durva treatment. This resulted in an imbalance between the Atezo/Bev and Treme/Durva and is potentially a limitation in comparing outcomes against a uniform treatment regimen. Our institution began combining ^90^Y with Atezo/Bev in early 2021 several months after first-line approval and, thus, accumulated more experience combining these therapies. The first patient to receive ^90^Y-Treme/Durva did not start until mid-2023, limiting our ability to compare retrospective outcomes between treatment regimens.

## 5. Conclusions

The combination of ^90^Y-ICI was safe, with improved target and overall response rates that exceeded ICIs alone and translated into a PFS benefit in patients, particularly those with BCLC-C disease achieving a target CR.

## Figures and Tables

**Table 1:** Demographics.

General	Cohort (*n* = 37)
Age, median (IQR)	64 (61–69)
Age, median (IQR)	64 (61–69)
Gender, male, *n* (%)	30 (81)
Declared race, *n* (%)	
Caucasian/White	15 (40)
African American/Black	14 (38)
Other	8 (22)
**Hepatology**	
Etiology, *n* (%)	
HCV	20 (54)
HCV ALD	5 (13)
HBV	4 (11)
Other	8 (22)
Child-Pugh score, *n* (%)	
A5	11 (30)
A6	23 (62)
B7	1 (3)
B8	2 (5)
Bilirubin (mg/dL), median (IQR)	0.7 (0.5–1.2)
Albumin (g/dL), median (IQR)	3.3 (3.2–3.8)
Platelets (10^3^/mL), median (IQR)	215 (154–283)
Esophageal varices at baseline, *n* (%)	9 (24)
Unable to assess varies	5 (14)
**HCC**	
Diagnosis date, range	12/28/2020–2/27/2024
AFP (ng/mL), median (IQR)	86 (9–2050)
AFP (ng/mL), ≥400, *n* (%)	14 (38)
BCLC staging, *n* (%)	
A	7 (19)
B	10 (27)
C	20 (54)
ECOG score, *n* (%)	
0	23 (62)
1	14 (38)
Solitary HCC, *n* (%)	20 (54)
Index tumor size (cm), median (IQR)	8.0 (6.0–12)
Cumulative tumor size (cm), median (IQR)	10.8 (7.5–15.2)
Macrovascular invasion, *n* (%)	14 (38)
Extrahepatic disease, *n* (%)	9 (24)
Prior LDT, *n* (%)	4 (11)
**Immune Checkpoint Inhibitors**	
ICI therapy	
Atezolizumab/Bevacizumab	30 (81)
Tremelimumab/Durvalumab	7 (19)
Treatment sequence lead	
ICI, *n* (%)	32 (86)
^90^Y, *n* (%)	5 (14)

**Abbreviations**: Interquartile range (IQR), Hepatitis C virus (HCV), Hepatitis B virus (HBV), Alcoholic liver disease (ALD), Alpha feto-protein (AFP), Barcelona Clinic Liver Cancer (BCLC), Eastern Cooperative Oncology Group (ECOG), Hepatocellular carcinoma (HCC), Liver-directed therapy (LDT), Immune checkpoint inhibitors (ICIs), Yttrium-90 (^90^Y).

**Table 2:** Immune checkpoint inhibitor and ^90^Y treatment.

ICI Therapy	Cohort (*n* = 37)
**Atezolizumab/Bevacizumab**	
Start date, range	1/22/2021–5/31/2024
Patients receiving Atezo/Bev therapy, *n* (% of total)	30 (81)
Number of cycles of Atezo, median (range)	10 (1–49)
Number of cycles of Bev, median (range)	4 (0–23)
Duration of Atezo/Bev, months, median (IQR)	8 (4–16)
Discontinuation, *n* (% of Atezo/Bev)	14 (47)
Time to discontinuation, months, median (IQR)	5 (2–7)
Switched to different systemic therapy, *n* (%)	6 (20)
**Tremelimumab/Durvalumab**	
Start date, range	3/24/2023–9/13/2023
Patients receiving Treme/Durva therapy, *n* (% of total)	7 (18)
Number of cycles of Durva, median (range)	9 (2–15)
Duration of Treme/Durva, months, median (IQR)	13 (6–18)
Discontinuation, *n* (% of Treme + Durva)	3 (43)
Time to discontinuation, months, median (IQR)	6 (1–8)
Switch to different systemic therapy, *n* (%)	2 (29)
**First Cycle ^90^Y Characteristics**	
First cycle ^90^Y date, range	3/9/2021–5/20/2024
Patients receiving ^90^Y, *n* (% of total)	37 (100)
Target perfusion volume (mL), median (IQR)	573 (274–978)
Target dose to volume (Gy), median (IQR)	472 (313–556)
Lung shunt fraction (%), median (IQR)	5.0 (3.3–8.1)
Multicycle ^90^Y, *n* (%)	11 (30)
Number of total ^90^Y treatments, median (range)	1 (1–4)
DEB-TACE bridge to ^90^Y	3 (8)
**Treatment Sequence Lead**	
ICI, *n* (%)	32 (86)
^90^Y, *n* (%)	5 (14)
Time from ICI lead -> ^90^Y, days, median (IQR)	48 (21–76)
Time from ^90^Y lead -> ICI, days, median (IQR)	18 (12–48)
DEB-TACE bridge to ^90^Y	3 (8)

**Abbreviations**: Yttrium-90 (^90^Y), Interquartile range (IQR), Gray (Gy), Doxorubicin eluting beads transarterial embolization (DEB-TACE), Immune checkpoint inhibitors (ICIs).

**Table 3:** Adverse events by treatment.

All AEs	Cohort (*n* = 37)
Any AEs, *n* of patients (% total)	33 (89)
Any grade 3 or 4, *n* of patients (% total)	6 (16)
AEs that led to discontinuation, *n* of patients (% total)	4 (11)
AEs that led to delay in treatment, *n* of patients (% of total)	9 (24)
AEs that led to death, *n* of patients (% of total)	0 (0)
Immune-mediated AE requiring steroid use, *n* of patients (% of total)	8 (22)
**Atezolizumab/Bevacizumab AEs**	**Cohort (*n* = 30)**
Duration of treatment, months, median (IQR)	8 (3–16)
Follow-up time, months (IQR)	15 (8–26)
Any, *n* of patients (%)	25 (83)
Any grade 3 or 4, *n* of patients (%)	5 (16)
AEs that led to discontinuation, *n* of patients (%)	4 (13)
AEs that led to delay in treatment, *n* of patients (%)	4 (13)
AEs that led to death, *n* of patients (%)	0 (0)
Immune-mediated AE requiring steroid use, *n* of patients (%)	5 (17)
**Tremelimumab/Durvalumab AEs**	**Cohort (*n* = 7)**
Duration of treatment, months, median (IQR)	13 (6–18)
Follow-up time, months (IQR)	16 (8–19)
Any, *n* of patients (%)	5 (71)
Any grade 3 or 4, *n* of patients (%)	0 (0)
AEs that led to discontinuation, *n* of patients (%)	0 (0)
AEs that led to delay in treatment, *n* of patients (%)	3 (43)
AEs that led to death, *n* of patients (%)	0 (0)
Immune-mediated AE requiring steroid use, *n* of patients (%)	3 (43)
**^90^** **Y AEs**	**Cohort (*n* = 37)**
Number of ^90^Y treatments, median (range)	1 (1–4)
Any, *n* of patients (% of total)	20 (54)
Any grade 3 or 4, *n* of patients (% of total)	0 (0)
AEs that led to discontinuation, *n* of patients (% of total)	0 (0)
AEs that led to delay in treatment, *n* of patients (% of total)	2 (5)
AEs that led to death, *n* of patients (% of total)	0 (0)
Immune-mediated AE requiring steroid use, *n* of patients (% of total)	0 (0)

**Abbreviations**: Adverse events (AEs), Immune checkpoint inhibitors (ICIs), Yttrium-90 (^90^Y).

**Table 4:** Response rates and outcomes to ICI-^90^Y.

Outcomes	
Time to follow-up (months), median (IQR)	16 (8–25)
Overall progression, *n* (% total)	21 (57)
Death, *n* (% total)	22 (59)
Unable to assess response, *n* (% of total)	1 (3)
**Target Response following ^90^Y-ICI**	
Target CR, *n* (% total)	18 (50)
Target ORR (CR/PR), *n* (% total)	30 (83)
**Overall Response following ^90^Y-ICI**	
Overall CR, *n* (% total)	14 (39)
Overall ORR (CR/PR), *n* (% total)	22 (61)
Time after ICI initiation to response rate, months, median (IQR)	3.4 (2.3–4.2)
**Outcomes**	
Time to follow-up (months), median (IQR)	16 (8–25)
Overall progression, *n* (% total)	21 (57)
Death, *n* (% total)	22 (59)
Unable to assess response, *n* (% of total)	1 (3)

**Abbreviations**: Adverse events (AEs), Immune checkpoint inhibitors (ICIs), Yttrium-90 (^90^Y).

**Table 5:** ICI-^90^Y treatment characteristics by BCLC stage.

	BCLC A-B	BCLC-C	*p* Value
Cohort, *n*	17	20	
**Hepatology**			
Etiology, *n* (%)			**0.006**
Viral	10 (59)	19 (95)	
Other	7 (41)	1 (5)	
Child-Pugh score, *n* (%)			0.451
A5/A6	15 (88)	19 (95)	
B7/B8	2 (12)	1 (5)	
Bilirubin (mg/dL), median (IQR)	0.8 (0.5–1.7)	0.7 (0.5–1.1)	0.602
Albumin (g/dL), median (IQR)	3.3 (3.2–3.9)	3.4 (3.3–3.8)	0.389
Platelets (10^3^/mL), median (IQR)	222 (134–271)	203 (180–300)	0.604
**HCC**			
AFP (ng/mL), median (IQR)	41 (5–101)	598 (55–32,066)	**0.009**
Solitary HCC, *n* (%)	7 (41)	13 (65)	0.146
Index tumor size (cm), median (IQR)	8.5 (5.3–12.1)	7.6 (6.1–10.9)	0.692
Cumulative tumor size (cm), median (IQR)	12 (8.2–15.5)	8.7 (7.0–15)	0.180
Macrovascular invasion, *n* (%)	0 (0)	14 (70)	
Extrahepatic disease, *n* (%)	0 (0)	9 (45)	
**Immune Checkpoint Inhibitors**			
Patients receiving Atezo/Bev therapy, *n* (% of total)	14 (82)	16 (80)	
Duration of Atezo/Bev, months, median (IQR)	10 (5–25)	6 (3–11)	0.073
Discontinuation, *n* (% of Atezo/Bev)	2 (14)	12 (75)	**<0.001**
Patients receiving Treme/Durva therapy, *n* (% of total)	3 (18)	4 (20)	
Duration of Treme/Durva, months, median (IQR)	16 (6–18)	11 (3–18)	0.658
Discontinuation, *n* (% of Treme/Durva)	1 (33)	2 (50)	0.724
**First Cycle ^90^Y Characteristics**			
Target perfusion volume (mL), median (IQR)	622 (384–1084)	458 (201–922)	0.111
Target dose to volume (Gy), median (IQR)	375 (206–537)	501 (415–562)	0.083
Lung shunt fraction (%), median (IQR)	6.1 (3.6–10.7)	4.5 (2.4–6.0)	0.070
Multicycle ^90^Y, *n* (%)	6 (35)	5 (25)	0.295
Number of total ^90^Y treatments, median (range)	2 (1–3)	1 (1–4)	0.599
DEB-TACE bridge to ^90^Y	3 (18)	1 (5)	0.211
**Treatment Sequence Lead**			
Sequence lead, ICI vs. ^90^Y lead	12 (71)	20 (100)	**0.003**
Time from ICI lead -> ^90^Y, days, median (IQR)	37 (14–74)	53 (25–80)	0.572
Time from ^90^Y lead -> ICI, days, median (IQR)	18 (12–48)	0	

**Abbreviations**: Yttrium-90 (^90^Y), Complete response (CR), Partial response (PR), Objective response rate (ORR), Adverse events (AEs), Immune checkpoint inhibitor (ICI).

## Data Availability

All data included in this study are available upon reasonable request to the corresponding author.

## Supplementary Materials

The following supporting information can be downloaded at: https://www.mdpi.com/article/10.3390/cancers17172745/s1, Table S1: All Adverse Events from ICI and ^90^Y; Table S2: Adverse Events by ICI Regimen and Sequence Lead; Table S3: Response Rates by ICI Regimen and Sequence Lead; Table S4: ^90^Y-ICI Treatment Characteristics by BCLC Stage.

## Abbreviations

The following abbreviations are used in this manuscript:

HCC	Hepatocellular carcinoma
ICI	Immune checkpoint inhibitors
TKI	Tyrosine-kinase inhibitors
OS	Overall survival
ORR	Objective response rate
LDT	Liver-directed therapy
BCLC	Barcelona Clinic Liver Cancer
TACE	Transarterial chemoembolization
TARE	Transarterial radioembolization
90Y	Yttrium-90
Atezo	Atezolizumab
Bev	Bevacizumab
Treme	Tremelimumab
Durva	Durvalumab
PFS	Progression-free survival
CR	Complete response
OR	Objective response
TTP	Time to progression
IQR	Interquartile range
