# Supplementary material for: Contemporary Clinical Utilization of Radioembolization with Immune Checkpoint Inhibitors as First-Line Treatment in HCC: Real-World Report on Safety and Outcomes
Source: Cancers (Basel). 2025 Aug 23;17(17):2745. doi: 10.3390/cancers17172745 (PMC12427573; doi:10.3390/cancers17172745)
Supplement: Supplementary file 1 [file cancers-17-02745-s001.zip › cancers-3804059-supplementary.pdf]

SUPPLEMENTAL TABLES

Supplemental Table S1. All Adverse Events from ICI and ^{90}Y				
	Cohort (n = 37)			
	ICI		^{90}Y	
**All Aes**	**Any Grade**	**Grade 3 or 4**	**Any Grade**	**Grade 3 or 4**
Fatigue, n (% of total)	11 (30)	0 (0)	9 (24)	0 (0)
AST increase, n (% of total)	1 (3)	0 (0)	2 (5)	0 (0)
ALT increase, n (% of total)	1 (3)	0 (0)	2 (5)	0 (0)
Platelet count decrease, n (% of total)	1 (3)	0 (0)	1 (3)	0 (0)
Decrease appetite, n (% of total)	3 (8)	0 (0)	13 (35)	0 (0)
Bilirubin increase, n (% of total)	1 (3)	0 (0)	0 (0)	0 (0)
Rash, n (% of total)	12 (32)	0 (0)	0 (0)	0 (0)
Hypertension, n (% of total)	10 (27)	0 (0)	0 (0)	0 (0)
Nausea, n (% of total)	1 (3)	0 (0)	10 (27)	0 (0)
Asthenia, n (% of total)	4 (11)	0 (0)	3 (8)	0 (0)
Vomiting, n (% of total)	3 (8)	0 (0)	5 (14)	0 (0)
Constipation, n (% of total)	6 (16)	1 (3)	0 (0)	0 (0)
Abdominal pain, n (% of total)	0 (0)	0 (0)	7 (19)	0 (0)
Edema, n (% of total)	6 (16)	1 (3)	0 (0)	0 (0)
Pruritus, n (% of total)	6 (16)	0 (0)	0 (0)	0 (0)
Weight decrease, n (% of total)	2 (5)	0 (0)	4 (11)	0 (0)
Pyrexia, n (% of total)	1 (3)	0 (0)	3 (8)	0 (0)
Diarrhea, n (% of total)	3 (8)	1 (3)	0 (0)	0 (0)
Cough, n (% of total)	3 (8)	0 (0)	0 (0)	0 (0)
Proteinuria, n (% of total)	1 (3)	0 (0)	0 (0)	0 (0)
Mucositis, n (% of total)	1 (3)	1 (3)	0 (0)	0 (0)
Epistaxis, n (% of total)	2 (5)	0 (0)	0 (0)	0 (0)
Insomnia, n (% of total)	1 (3)	0 (0)	0 (0)	0 (0)
Pneumonitis, n (% of total)	0 (0)	0 (0)	0 (0)	0 (0)
Bowel perforation, n (% of total)	0 (0)	2 (5)	0 (0)	0 (0)
Anemia, n (% of total)	1 (3)	0 (0)	0 (0)	0 (0)
Arthralgias, n (% of total)	1 (3)	0 (0)	0 (0)	0 (0)
Myasthenia Crisis, n (% of total)	0 (0)	1 (3)	0 (0)	0 (0)
**Abbreviations**: Adverse events (AEs), Aspartate aminotransferase (AST), Alanine aminotransferase (ALT).				

**Supplemental Table S2. Adverse Events by ICI Regimen and Sequence Lead**			
**ICI Regime - AEs**	**Atezo/Bev**	**Treme/Durva**	**P Value**
Number of patients	30	7	
Any AEs, n of patients (% total)	25 (83)	5 (71)	0.131
Any grade 3 or 4, n of patients (% total)	5 (16)	0 (0)	0.132
AEs that led to discontinuation, n of patients (% total)	4 (13)	0 (0)	0.181
AEs that led to delay in treatment, n of patients (% of total)	4 (13)	3 (43)	0.186
AEs that led to death, n of patients (% of total)	0 (0)	0 (0)	
Immune-mediated AE requiring steroid use, n of patients (% of total)	5 (17)	3 (43)	0.153
**Sequence Lead - AEs**	**ICI Lead**	**^{90}Y Lead**	**P Value**
Number of patients	32	5	
Any AEs, n of patients (%)	30 (94)	3 (60)	0.056
Any grade 3 or 4, n of patients (%)	5 (16)	0 (0)	0.210
AEs that led to discontinuation, n of patients (%)	4 (13)	0 (0)	0.267
AEs that led to delay in treatment, n of patients (%)	8 (27)	1 (20)	0.168
AEs that led to death, n of patients (%)	0 (0)	0 (0)	
Immune-mediated AE requiring steroid use, n of patients (%)	8 (27)	0 (0)	0.104
**Abbreviations**: Adverse events (AEs), Immune checkpoint inhibitors (ICI),Yttrium-90 (^{90}Y), Atezolizumab (Atezo), Bevacizumab (Bev), Tremelimumab (Treme), Durvalumab (Durva).			

Supplemental Table S3. Response Rates by ICI Regimen and Sequence Lead			
**Response Rate by ICI Regimen**	**Atezo/Bev**	**Treme/Durva**	**P Value**
Number of patients	29	7	
Target CR, n (%)	14 (48)	4 (57)	0.673
Target ORR (CR/PR), n (%)	23 (79)	7 (100)	0.090
Overall CR, n (%)	11 (40)	3 (43)	0.811
Overall ORR (CR/PR), n (%)	16 (55)	5 (71)	0.426
**Response Rate by Sequence Lead**	**ICI Lead**	**^{90}Y Lead**	**P Value**
Number of patients	32	5	
Target CR, n (%)	15 (48)	3 (60)	0.629
Target ORR (CR/PR), n (%)	27 (87)	3 (60)	0.172
Overall CR, n (%)	11 (35)	3 (60)	0.303
Overall ORR (CR/PR), n (%)	18 (58)	3 (60)	0.935

Abbreviations: Adverse events (AEs), Immune checkpoint inhibitors (ICI),Yttrium-90 (^{90}Y), Atezolizumab (Atezo), Bevacizumab (Bev), Tremelimumab (Treme), Durvalumab (Durva).

**Supplemental Table S4. ^{90}Y-ICI Treatment Characteristics by BCLC Stage**			
	**BCLC- A-B**	**BCLC-C**	**P Value**
Cohort, n	17	20	
**AEs**			
Any AEs, n of patients (% total)	14 (82)	19 (95)	0.211
Any grade 3 or 4, n of patients (% total)	2 (12)	3 (15)	0.773
AEs that led to discontinuation, n of patients (% total)	1 (6)	3 (15)	0.361
AEs that led to delay in treatment, n of patients (% of total)	4 (24)	5 (25)	0.979
AEs that led to death, n of patients (% of total)	0	0	
Immune-mediated AE requiring steroid use, n of patients (% of total)	4 (24)	4 (20)	0.795
**Response to ^{90}Y-ICI**			
Unable to assess response, n (% total)	1 (6)	0 (0)	
**Target Response following ^{90}Y-ICI**			
Target CR, n (% total)	8 (50)	10 (50)	1
Target ORR (CR/PR), n (% total)	13 (81)	17 (85)	0.765
**Overall Response Following ^{90}Y-ICI**			
Overall CR, n (% total)	7 (44)	7 (35)	0.593
Overall ORR (CR/PR), n (% total)	11 (69)	10 (50)	0.254

Abbreviations: Yttrium-90 (^{90}Y), Complete response (CR), Partial response (PR), Objective response rate (ORR), Adverse events (AEs), Immune checkpoint inhibitor (ICI).
